# The Influence of DHA on Language Development: A Review of Randomized Controlled Trials of DHA Supplementation in Pregnancy, the Neonatal Period, and Infancy

**DOI:** 10.3390/nu12103106

**Published:** 2020-10-12

**Authors:** Nicola R. Gawlik, Amanda J. Anderson, Maria Makrides, Lisa Kettler, Jacqueline F. Gould

**Affiliations:** 1 Discipline of Paediatrics, Faculty of Health and Medical Sciences, The University of Adelaide, North Terrace, Adelaide 5000, Australia; nr.gawlik@rasa.org.au (N.R.G.); mandy.anderson@adelaide.edu.au (A.J.A.); maria.makrides@sahmri.com (M.M.); 2 Women and Kids, South Australian Health and Medical Research Institute, 72 King William Road, North Adelaide 5006, Australia; 3 Trinity College Gawler Inc., Alexander Avenue, Evanston South 5116, Australia; kettleli@trinity.sa.edu.au; 4 School of Psychology & Discipline of Paediatrics, Faculty of Health and Medical Sciences, The University of Adelaide, North Terrace, Adelaide 5000, Australia

**Keywords:** DHA, omega-3 fatty acids, supplementation, language, verbal abilities, speech, prenatal, postnatal, neonatal, infant

## Abstract

This review summarizes randomized controlled trials (RCTs) assessing the effect of docosahexaenoic acid (DHA) supplementation in the first 1000 days on child language. Six databases were searched and RCTs were included if they involved supplementation with DHA during pregnancy, to preterm infants, or during the postpartum period, included a placebo group with less or no DHA, and reported a language outcome. We included 29 RCTs involving *n* = 10,405 participants from 49 publications. There was a total of 84 language measures at ages ranging from 3 months to 12 years. Of the 84 assessments, there were 4 instances where the DHA group had improved scores, and 2 instances where the DHA group had worse scores (with the majority of these significant effects found within one RCT). The remaining comparisons were null. A few RCTs that included subgroup analyses reported (inconsistent) effects. There was limited evidence that DHA supplementation had any effect on language development, although there were some rare instances of both possible positive and adverse effects, particularly within population subgroups. It is important that any subgroup effects are verified in future trials that are adequately powered to confirm such effects.

## 1. Introduction

The first 1000 days of life is a period of rapid brain development where embryonic stem cells develop into a functioning brain that is 80% of the size of an adult brain [1]. Although the brain continues to develop well into early adulthood, the foundations are laid for later development during this critical period [2]. Appropriate nutrition in early life is considered to be one of the most important (non-genetic) influences on brain development [2,3,4,5,6,7]. The omega-3 long chain polyunsaturated fatty acid, docosahexaenoic acid (DHA, 22:6n−3) is one nutrient that is concentrated in neural tissues, and is actively accumulated in the brain during early development [8,9,10].

Observational studies have demonstrated associations between intake of foods rich in DHA (oily fish) with positive outcomes for child development [11,12,13,14,15,16,17,18]. A number of randomized controlled trials (RCTs) have been conducted to further explore whether increasing exposure to DHA through supplementation in the first 1000 days can enhance brain development. Cochrane reviews and meta-analyses of these RCTs have revealed mixed and predominantly null effects of DHA interventions on brain development [19,20,21,22,23]. RCTs have predominantly evaluated brain development through assessments of cognition, however evidence is emerging to suggest that language may be a specific domain of brain development sensitive to early DHA exposure [14,15,16,17,24,25,26,27,28,29,30,31,32].

### 1.1. The Development and Assessment of Language Abilities

Language is a critical component of overall development during infancy and childhood. Language skills are necessary for understanding and communicating with others, forming relationships with others, thinking and problem solving, learning, reading, and writing [33,34]. Language abilities develop rapidly across childhood and are categorized as either receptive or expressive. Receptive language abilities refer to receiving, internal processing, and understanding the meaning of information received from another person. Expressive language abilities refer to an individual’s ability to express and convey information to another person. Receptive language begins to develop before birth and young infants can recognize the speech patterns of their mother’s voice from exposure during pregnancy. By around six months of age, an infant can recognize the voice of their primary caregivers, the basic characteristic sounds of their caregiver’s native language, and other important or regular sounds in their environment. During this same period of infancy, expressive language emerges with infants crying to express discomfort, or needs such as hunger, and later starting to smile, laugh, and babble. Over the next year, infants begin to recognize and understand some commonly used words and start to speak individual words, with meaning. Across early childhood the number and complexity of words and sentences understood and spoken increases. By school age, children can form sentences with appropriate structure, word endings, tense, and auxiliaries.

Language assessments can be based on caregiver report or clinician administered and can either capture overall general language abilities or a specific domain (such as only receptive or only expressive language abilities). As language abilities emerge and develop across childhood [35], assessments of language during infancy and childhood must be developmentally appropriate for the age of the child. Assessments during infancy and early childhood are often based on caregiver report, generally requiring caregivers indicate from lists words or phrases used (expressive language) or understood (receptive language, also labeled as verbal comprehension in many assessments) in daily life by the child. In older children, global cognitive (clinician administered) tests, such as those measuring Intelligence Quotient, include a Verbal Intelligence Quotient (VIQ), or equivalent, that captures acquired verbal knowledge, and language-based cognitive reasoning abilities. Tests of academic abilities typically include an assessment of language-dependent skills reading and spelling [33,34]. Caregiver reports have the advantage of comprehensively capturing the breadth of a child’s vocabulary, but are limited by the potential bias from caregiver report. Assessments administered by a clinician test whether a child recognizes, speaks, or understands particular standardized words, phrases or instructions in the assessment setting. The strength of clinician administered assessments is the standardized administration and absence of bias that may be associated with parental report; however, such assessments can only reflect performance on the day of assessment. Many assessments have age-normed references that indicate whether the performance of an individual child is appropriate for a typical child of the same age. Most normed tests have scores that are age-standardized to a mean of 100, and standard deviation of 15 so that performance falling below 85 indicates a possible language delay or impairment.

### 1.2. The Effect of DHA on Language Development

Initial support for the role of DHA in language development came from observational studies of consumption of fish and seafood (naturally rich sources of DHA) during pregnancy. The most compelling data linking maternal DHA intake during pregnancy with neurological benefits for the offspring comes from a high-quality cohort study of 5449 mother-child pairs (the Avon Longitudinal Study of Pregnancy and Childhood) [17]. The authors found that fish and seafood intake above the level recommended by the US government (340 g seafood/week) was associated with a decreased risk of being in the lowest quartile for VIQ at 8 years of age [17]. These results are supported by other smaller cohort studies reporting that increased fish and seafood intake during pregnancy is associated with more advanced vocabulary comprehension at 18 months [14], receptive vocabulary at 3 years [15], language development at 4 years [13], and VIQ at 9 years [16]. A study compared outcomes of infants born preterm (<37 weeks’ completed gestation) who received a routine lipid emulsion containing fish oil, to those born prior to this practice change who received a soy-based lipid emulsion [36]. Infants receiving the emulsion with fish oil were slightly less likely to have language impairment at 18–24 months of age than infants who received the soy-based lipid emulsion [36]. In infants, DHA intake in the first 6 months after birth through DHA fortified infant formula had higher VIQ at 4 years of age than infants fed formula without DHA fortification [37]. Conversely, a negative association between DHA status at 9 months of age has been reported with communication skills at 3 years of age, within girls only [38]. In each of these observational studies, whilst several aspects of child development, such as cognition, motor and behavior have been assessed at a range of ages, the strongest evidence for a positive association between DHA intake and development has consistently been in language outcomes [13,14,15,16,17,37]. However, observational studies are unsuitable for establishing causality due to the difficulty in adjusting for confounding factors that also influence language development [39]. Hence RCTs are necessary to determine whether there is an effect of increased DHA exposure on language development.

RCTs of DHA supplementation during the first 1000 days have been conducted during pregnancy [19,20], with infants born preterm [21,40], during breastfeeding for full-term infants [22], and with infants [23]. The largely null results from these RCTs may be partially attributable to the focus on cognitive development [19,20,21,22,23,40]. Although aspects of language are captured by various assessments in a many of these RCTs, these are often overlooked as secondary or exploratory outcomes, and hence there is yet to be a review where language outcomes are collated and considered as a whole [19,20,21,22,23,40,41]. Furthermore, whilst adequate DHA is likely to be important throughout the whole of the first 1000 days, two reviews to date have attempted to synthesize [42] or provide at least a very brief overview [43] of the evidence across this period and language was not reported in either.

Here we aim to gain an overview of the totality of the evidence around the effect of early DHA supplementation on language development in childhood. We review RCTs with a DHA intervention at any period during the first 1000 days that include a measure of language, or any assessments of language-based cognitive or academic abilities.

## 2. Materials and Methods

This review was conducted according to the Preferred Reporting Items for Systematic Reviews and Meta-Analysis (PRISMA) guidelines [44].

### 2.1. Search Strategy

Six electronic databases were searched; Cumulative Index to Nursing and Allied Health Literature (CINAHL), Cochrane Central Register of Controlled Trials (CENTRAL) Current Contents Connect, Excerpta Medica dataBASE (EMBASE), PsycINFO, PubMed, and Web of Science. Strategies tailored to each database were based on the PubMed search; “DHA OR Docosahex*enoic acid OR docosahex*enoate OR omega 3 OR LCPUFA OR long chain polyunsaturated fatty acid OR fish oil OR marine oil OR algal oil” AND “Language OR linguistics OR verbal OR vocabulary OR literacy OR reading OR communication OR language test OR neurodevelopment OR cognitive development” AND “RCT OR randomi*e* OR intervention OR placebo OR control”. The search strategy for other databases is available from the authors, upon request. No date restrictions were set although studies had to be published in a journal and in English, and searches were limited to trials on humans. The titles and abstracts of all articles retrieved by the search were screened to assess eligibility. The reference lists of eligible articles identified by the search were also checked for other potentially relevant articles. In addition, we searched the reference lists of relevant reviews [19,20,21,22,23,40]. Search engines used were set up to email new publications identified by the search on a monthly basis, with new articles added to the review up until acceptance of the manuscript.

### 2.2. Inclusion and Exclusion Criteria

To be eligible for inclusion, a trial had to be in English, be conducted in humans, have a RCT design, include supplementation with DHA (where trials that included long-chain polyunsaturated fatty acids (LCPUFAs) in conjunction with DHA were also considered), include a placebo group without, or with less, DHA, with the intervention occurring during pregnancy, the postpartum period, or during infancy, and report a language outcome.

### 2.3. Data Extraction

Three authors were involved with reviewing search results and extracting data from included articles (J.F.G., N.R.G., and A.J.A.). Where relevant data or details were absent in an article, study authors were contacted. Study characteristics extracted included authors, publication year, sample information, intervention details, and language assessment and results. Possible sources of bias in each trial were noted.

### 2.4. Data Synthesis

Included studies were grouped according to the intervention period as (1) maternal prenatal interventions; (2) interventions for preterm infants; (3) postnatal interventions for breastfeeding mothers; and (4) postnatal interventions for infants. Although preterm neonates are typically supplemented during the same period of brain development as in trials where supplementation took place prenatally, they are best considered separately. Infants born preterm undergo their fetal brain growth spurt ex-utero and, hence, often have different developmental characteristics to term-born infants, such as poorer cognitive development and increased risk of behavioral problems [45,46,47,48,49,50]. Interventions that commenced either during pregnancy, or to preterm infants and continued through to infancy, were considered to be prenatal and preterm infant interventions, respectively.

Data were extracted into a table of characteristics of the included RCTs with the overall language assessment results. In order to comprehensively capture the language development of young children, language outcomes derived from multiple sources were included. Results of language assessments were categorized and discussed as:

(a) Clinician-administered global language measures (including language-specific subscales of global developmental tests);
(b) Parent-rated assessments of global language or domain specific language abilities (including language, communication or verbal scores from parent-completed assessments of non-language domains such as global development or behavior;
(c) Assessments of language-based cognitive abilities;
(d) Language-based academic abilities;
(e) Other language measures not classified above, particularly experimental measures;
(f) Subgroup effects (where reported) such as for sex, socio-economic status or birthweight.

## 3. Results

The initial search resulted in 1304 articles, 634 of which remained after duplicates were removed. Article abstracts were reviewed and 593 were excluded for the following reasons: not human trials (*n* = 63), did not involve omega-3 LCPUFA supplementation (*n* = 127), were not RCTs (*n* = 302) or did not have a language outcome (*n* = 101). The full text of 41 citations were then examined in detail and further exclusions were made primarily due to a focus on the effects of supplementation outside the first 1000 days (*n* = 16). Subsequent searches identified 13 subsequent eligible studies. A total of 29 studies were identified as eligible for inclusion, with relevant trial outcomes or details published across 49 articles.

### 3.1. Study Characteristics

There was a total of *n* = 10,405 participants included between the 29 studies (see Table 1). The trials and follow-up results were published between 1998 and 2020 and were predominantly conducted in high-income countries (the United States of America (USA), Australia, Canada, Sweden, Denmark, the United Kingdom (UK), the Netherlands, Belgium and Italy), with three in low- or middle-income countries (Iran, Mexico, and Ethiopia) [26,51,52,53]. English [54] Andrew, 2018 #3268; Devlin, 2017 #3272; Carlson, 2013 #3273; Colombo, 2019 #3067; O’Connor, 2001 #37; Fewtrell, 2002 #2761; Fewtrell, 2004 #2762; Isaacs, 2011 #507; Makrides, 2009 #33; Smithers, 2010 #825; Collins, 2015 #1693; Keim, 2018 #3110; Miller, 2016 #3111; Keenan, 2014 #2391; Keenan, 2016 #2389; Mulder, 2014 #2400; Mulder, 2018 #2916; Dunstan, 2008 #62; Meldrum, 2015 #1775; Makrides, 2010 #415; Makrides, 2014 #1564; Gould, 2017 #2559; Jensen, 2005 #3128; Jensen, 2010 #642; Meldrum, 2012 #3127; Scott, 1998 #146; Auestad, 2001 #2295; Auestad, 2003 #25; Birch, 2000 #116; Birch, 2007 #3115; Lucas, 1999 #3130; Willatts, 2013 #2402; Colombo, 2013 #2291; Drover, 2011 #2293; Drover, 2012 #680}, Swedish [55], Danish [32], Norweigen [56,57], German [58], Farsi [26], Spanish [51,52], Dutch [59,60], Flemish [61], and Italian [61] were the languages spoken. Most children participating in the trials were from singleton pregnancies although some included twin [54,62,63,64,65,66,67,68,69,70] and triplet [65,66,67,68,69,70] pregnancies. Three prenatal trials did not report whether multiple pregnancies were included in outcome assessments [56,57,71,72,73,74]. Three trials in pregnancy and one postnatal trial for infants were restricted to populations with a history of allergic disease [55,74,75,76].

### 3.2. Participants and Intervention

#### 3.2.1. Maternal Prenatal Interventions

Ten RCTs have investigated the effect of maternal prenatal supplementation in *n* = 4894 infants [25,26,27,28,51,52,58,75,76,77,78,79,80,81,82,83]. Some trials included exclusively term infants in their follow-ups [27,28,55,74,75,76,83] whereas one RCT specifically included preterm as well as term infants [25,77,78], as shown in Table 1.

Mothers took capsules with DHA doses of 120 [26], 300 [83], 400 [27,28,51,52], 450 [81,82], 600 [79,80], 800 [25,77,78], 1020 [58], 1100 [55], and 2200 [75,76] mg per day. Trials generally examined the effect of DHA in combination with eicosapentaenoic acid (EPA). The supplementation period commenced between mid-pregnancy and ended at birth in most trials [25,27,28,51,52,75,76,77,78,79,80,81,82]. Some trials continued the intervention after birth to 30 days [26], 3 months [83], 3–3.5 months [55], and 4 months [58]. Intervention after birth was through infant formula and supplements for breastfeeding mothers.

#### 3.2.2. Interventions for Preterm Infants

There were 8 RCTs that supplemented *n* = 2214 preterm infants [24,54,56,57,64,65,66,67,68,69,70,84,85]. The intervention was administered to infants through supplements for breastfeeding mothers, supplemented infant formula, through enteral feeds, or as sprinkles or a dissoluble powder that could be added to milk or food. It is noteworthy that in some trials, interventions in exclusively formula-fed infants compared formulas containing some DHA, to formulas that contained no DHA [64,65,66,67]. Other trials compared a low or standard dose of DHA to a higher dose of DHA [54,56,57,68,69,70,84]. Only one trial comparing low-dose DHA to high-dose DHA, included breastfed babies as well as formula fed babies [68,69,70]. All but one study [24] commenced the intervention within the first week of birth. Three of the trials finished the intervention by the time the infant was discharged from hospital (at around term-equivalent age) [54,56,57,65,68,69,70,84]. Three trials continued the intervention past discharge, until infants were aged 9 [66,67], 12 [64], or 24 months of age [85]. One trial administered the intervention later in infancy, commencing at 10 to 16 months of age, once formula feeding and breastfeeding had ceased [24]. The doses administered were largely dependent on the amount of breastmilk or formula the infants were able to consume on a daily basis and hence varied within intervention groups.

#### 3.2.3. Postnatal Interventions for Breastfeeding Mothers

Three trials assessed DHA interventions for breastfeeding women in *n* = 765 mother-infant pairs [32,53,62,63]. In one trial mothers were provided with capsules, and in another trial mothers had the option of capsules, muesli bars, or cookies with the supplementation period commencing within 5 days of birth and lasing up until 4 months of age [32,62,63]. The dosage of daily DHA was 200 mg in one of these trials [62,63], and was not reported in the other trial [32]. One trial targeting women with habitual low fish intake (for the population) additionally included a reference group of non-randomized breastfeeding women with high habitual fish intake [32]. The third trial involved randomizing breastfed infants aged 6 to 12 months to 4 intervention groups for a 12-month intervention [53]. In this study, mothers were provided with capsules containing 215 mg DHA, and infants were provided with a corn–soy blend complimentary food supplement that included 19 micronutrients (with or without 285 mg DHA depending on randomization group) [53]. One group received study products containing DHA for mothers only, one group received DHA study products for both mothers and children, one group received DHA in the child supplement only and the fourth group received capsules and child complimentary food supplements devoid of DHA [53]. The four groups were compared individually, rather than combined. This trial was conducted in Ethiopia where dietary DHA intake is reported to be habitually low [53].

#### 3.2.4. Postnatal Interventions for Infants

There were eight RCTs involving *n* = 2532 infants [29,30,31,59,60,61,71,72,73,74,86,87,88,89,90]. Only two trials included infants that were breastfed [74,90] and the majority included only term-born infants [29,30,31,59,60,71,72,73,74,86,87,88,90]. One RCT provided fish oil or olive oil capsules that were added to formula or breastmilk [74], one provided sprinkles to add to food [90], and six RCTs compared infant formula supplemented with DHA to infant formula devoid of DHA [29,30,31,59,60,61,71,72,73,86,87,88]. Infants received doses of DHA ranging from 0.12 to 0.96% of total fatty acids present in formula [29,30,31,59,60,61,71,72,73,86,87,88], or 250 to 280 mg DHA as oil per day [74], or up to 200 mg/d through sprinkles [90]. RCTs examined the effect of DHA alone, in combination with arachidonic acid (AA), or EPA, or both AA and EPA. Three trials involved more than one treatment group with differences between groups being source and dosage of DHA as well as combination with other fatty acids [29,30,31,71,72,73,87,88]. One formula trial included a breastfed group as a comparative reference group [59]. The supplementation period commenced within the first week of life and went for at least four months and up until a maximum of 12 months of age [29,30,31,59,60,61,71,72,73,74,86,87,88], or started at 12–14 months up until 24 months in one trial [90].

### 3.3. Possible Sources of Bias

Trials generally had adequate sequence generation, treatment allocation and blinding of participant processes. One trial did not appear to adequately blind participants as 92.9% of participants correctly guessed their infant’s group allocation [74]. In this trial as well as one other there was higher attrition from the treatment group compared with the control group [74,75]. There was some evidence of reporting bias, where one trial with a relevant assessment was reported in an abstract in 2010 and is yet to be published in full [55], although other outcomes from this trials have been published [91,92].

Follow-up rates and attrition varied between the studies. Only five trials [25,64,65,66,67,68,69,70,77,78] had a follow-up rate of ≥80% which is the minimum follow-up considered acceptable for minimizing attrition bias [93].

### 3.4. Assessments of Language Abilities

Child language was assessed with a total of 83 assessments at a variety of different ages between 3 months [82] and 12 years [76].

(a) Clinician-administered global language measures (including language-specific subscales of global developmental tests).

There were 16 studies that used a clinician-administered test specifically designed to assess language or a clinician-administered global development assessment with a subscale capturing overall language abilities; five prenatal trials [25,28,75,77,78,82,83], five preterm infant trials [24,54,65,66,85], 2 postnatal trials for breastfed infants [53,62], and six postnatal trials for infants [29,30,31,73,74,86,87,89,90] Of these 16 studies, there were 10 language-specific assessments [28,29,31,62,73,75,77,78,80,89] and 16 global developmental assessments with an overall language subscale [24,25,28,30,53,54,65,66,74,75,82,83,85,86,87,90].

The Peabody Picture Vocabulary Test (PPVT), as used in five trials, [28,29,31,73,75,80] is designed to measure receptive vocabulary for individuals 2.5 years an older. The Clinical Evaluation of Language Fundamentals (CELF) assesses general language ability and included studies used editions for preschool children [77] as well as school aged-children [78,89]. The Clinical Linguistic and Auditory Milestone Scale (CLAMS) provides a language development quotient that was used in one trial at 2 ages, but with limited details available about the assessment [62].

As language is a key ability that emerges in early childhood, many general developmental tests designed to detect developmental delays in infants and toddlers specifically include a measure of early overall language development. The Bayley Scales of Infant Development (Bayley) is one of the most widely used development assessments for young children. It was developed for infants and toddlers (up to ~3.5 years of age) and has multiple editions. The Bayley-III was used in nine studies [24,25,28,54,74,82,83,85,90] and includes a specifically designed, standardized global Language Scale score made up of a receptive and expressive language score. Bayley editions I and II combine cognitive and language development into one mental scale although authors in 2 trials calculated a language score from this (which has been included in this review) [30,87]. The Griffiths Mental Development Scale (GMDS) [75] is a global developmental test from birth to 8 years that includes a Speech and Hearing subscale that captures expressive and receptive language. The Knobloch, Passamanick and Sherrard’s Developmental Screening Inventory (KPSDSI) is a global developmental assessment for infants and young children with a language subscale that was used in three trials for infants aged 9 months [65,66,86]. The Denver Developmental Screening Test (Denver) is a global developmental test that includes a language subscale. The Denver test was culturally adapted for use in Ethiopia and included items that were clinician administered as well as parent-report items [53].

(b) Parent-rated assessments of global language or domain-specific language abilities (including language, communication or verbal scores from parent-completed assessments of non-language domains, such as global development or behavior).

There were 12 trials reporting a parent-rated global or domain specific language assessment; five in prenatal trials [26,28,58,75,76,80], three in preterm infants [57,64,69], one in breastfeeding mothers [32], and in three postnatal trials in infants [30,71,72,74,89]. There were 11 parent-completed assessments specifically targeting language [28,30,32,64,69,71,72,74,76,80,89] and five assessments of another neurological domain that included a language-related score [26,57,58,74,75].

There were two parent-rated measures designed specifically to measure language abilities. The MacArthur-Bates Communicative Development Inventories (MCDI) are age-standardized forms to capture developmentally appropriate comprehension, non-verbal communication, vocabulary and early emergence of grammar depending on the age of the child. The MCDI was used in nine studies and involves a Words and Gestures form for children 8–18 months and a Words and Sentences form for children age 16–30 months [28,30,32,64,69,71,72,74,80]. The Children’s Communication Checklist (CCC) likewise specifically targets language and was developed as a screen for communication difficulties (both expressive and receptive) and pragmatic impairments in children aged 4 to 16 years and was used in two trials [76,89].

There were two general developmental questionnaires that included a language outcome, and one behavior questionnaire that had a language survey. The Child Development Inventory (CDI) is a parent-rated measure of global child development between the ages of 1 and 6 years. Along with an overall developmental score, there are subscales for Expressive Language, Language Comprehension, and Letters. A German-translation was used in one of the included trials [58]. The Ages and Stages Questionnaire (ASQ) is also designed as a global measure of general development for children aged 1 to 66 months. A subscale for Communication is included and was reported in two trials [26,57]. The Child Behavior Checklist (CBCL) is a measure of behavioral development and behavior problems with editions for young children (1.5 to 5 years) and older children (6–18 years). The questionnaire for young children includes a Language Development Survey where parents indicate the number words in their child’s vocabulary and was used in 2 of the include trials [74,75].

(c) Assessments of language-based cognitive abilities.

The majority of trials that evaluate the effect of a DHA intervention on neurodevelopment include a test of cognition, and many of these tests include an assessment of language-based cognitive abilities. We identified 12 trials that reported the results of a language-based cognitive ability; five prenatal trials [52,55,76,77,78,80], two trials in preterm infants [67,70], 1 in breastfeeding mothers [63], and in four postnatal interventions for infants [29,60,61,88]. 

Three IQ tests commonly used in child and young-adult populations were outcome assessments in the included trials; the Wechsler Abbreviated Scale of Intelligence (WASI) was used in five trials [60,67,70,78,84], the Wechsler Preschool and Primary Scale of Intelligence (WPPSI) was administered in six trials [29,55,61,63,80,88], and the Wechsler Intelligence Scale for Children (WISC) was conducted in one trial [76]. All include a Verbal Comprehension Index (an equivalent of a VIQ score) capturing crystallized or verbal abilities. The Differential Ability Scales (DAS) is an assessment of global cognitive functioning ability, similar to an IQ test. The DAS includes a Verbal Scale Score that measures verbal-based cognitive abilities [77]. The McCarthy Scales of Children’s Abilities (MSCA) is a cognitive development assessment for young children with a Verbal subscale that was used at 5 years in one prenatal trial [52]. The Developmental NEuroPSYchological Assessment (NEPSY) is a general cognition test battery for school-age children with a language domain that was used in two trials [60,67].

(d) Language-based academic abilities.

There were five reports of a language-based academic assessment; two in prenatal trials [78,80], two in preterm infants [67,70], and one in postnatally supplemented infants [31]. All language-based academic assessments were administered by a clinician. The Wide Range Achievement Test (WRAT) includes a Word Reading task and a Spelling task and was performed in one prenatal trial [78] and one preterm infant trial [70]. The Wechsler Individual Achievement Test (WIAT) is an educational assessment with a test each of Word Reading, Spelling, and Pseudoword decoding that was administered in one preterm infant trial at 10 years [67]. The Test of Preschool Early Literacy (TOPEL) is designed to detect early literacy problems in preschool aged children. The TOPEL includes subtests of vocabulary, phonological awareness, and print knowledge (knowledge of written language) and was used in one trial to assess 42-month-old infants [80]. The revised version of the Bracken Basic Concept Scale (BBCS) similarly assesses language-based abilities associated with school-readiness [31]. The BBCS was administered to 2.5-year-old infants to capture acquisition of basic concepts and aspects of receptive language [31].

(e) Other language measures not classified above, particularly experimental measures.

There were four trials that included an alternate, or more experimental measure of language. One trial measured mean length of utterances (MLU) at 39 months of age [73]. A mother and child were recorded during ~15–30 min of conversational free-play. Taped sessions were transcribed and coded to obtain the MLU score. One trial administered the Renfrew Bus Story at 6 years of age [89]. This task required the children to retell a narrative with correct sentence length, complexity and vocabulary. A prenatal trial assessed Sentence Repetition at 36, 42 and 48 months of age [80]. Children were required to repeat verbal sentences verbatim, despite increasing length and complexity [80]. One trial administered a Recognition task requiring the infants to react to English and non-English consonants at 9 months [27] and a word-object pairing task to the same infants at age 16 months of age [28] that were not otherwise described.

### 3.5. Efficacy of Intervention

#### 3.5.1. Assessments of Language Abilities after Maternal Prenatal DHA Intervention

The 10 trials of maternal prenatal DHA supplementation showed no overall significant differences between treatment and control group language development between 18 months and 7 years of age using 35 different assessments of language abilities [25,26,27,28,51,52,55,58,75,76,77,78,79,80,81,82,83,94].

(a) Of the 10 trials with prenatal interventions, there were four that conducted an assessment of language through a clinician [28,75,77,78,80]. The PPVT was administered at 2.5 years [75], 60 months [80] and 5–6 years of age [28] with no evidence of an effect of DHA supplementation at any age. The CELF-P2 was conducted with 4-year-old children [77] and then the CELF-4 was used with the same children at 7-years of age, with no effect of DHA intervention detected [78].

There were an additional 5 trials that included a global developmental assessment that contained an assessment of overall language abilities [25,28,75,82,83]. The Bayley-III language subscale was administered at 3 months [82], 4 months [83], 12 months [83], and 18 months [25,28] of age, between four RCTs with null effects reported. The GMDS speech and hearing subscale revealed no group differences at 2.5 years of age [75].

(b) Of the 10 RCTs conducted during pregnancy, there were three that included a parent-rated measure of language [28,76,80]. The MCDI was completed three times by parents, once at 14 months [28] and in two trials at 18 months [28,80] of age with no evidence of a benefit of prenatal DHA supplementation. The CCC was administered at 12 years of age with no group difference [76].

There were four parent-rated measures of development in general [26,58], and one behavioral questionnaire that included a language subscale [75]. The CDI was completed by parents at 4 years and again at 5 years of age, with no group differences in expressive language or language comprehension [58]. The general development ASQ was completed by parents at 4 months and 6 months of age and similarly revealed no group effects [26]. A language survey included in a behavior questionnaire completed when children were 2.5 years old showed no effect of DHA intervention [75].

(c) There were six IQ or cognitive tests with a VIQ or equivalent score reported between the 10 prenatal trials [55,76,77,78,80]. The WPPSI-III was administered at 46 months of age in one study [55] and at 48 months of age in another [80], with no evidence of an effect found in either. The DAS-II was administered at 4 years of age [77], and the WASI-II was administered to these same children at 7 years of age [78] with no effect of prenatal DHA supplementation reported. The WISC-IV was conducted at 12 years of age with no group differences found [76]. The MSCA administered at 5 years of age likewise detected no difference in group scores [52].
(d) Two out of the 10 prenatal DHA trials assessed language-based academic abilities. One trial assessed preschool aged children [80], and the other trial assessed 7-year-old children [78]. No differences were found with these academic measures of reading, spelling, vocabulary, phonological awareness, or knowledge of written language [78,80].
(e) Two of the 10 prenatal studies included an alternate/experimental assessment of language [27,28,80]. In one trial, the assessment was repeated at three ages [80], whilst the other trial used differing alternative language measures at different ages [27,28]. Neither trial found a group difference on any of the measures at any of the ages [27,28,80].
(f) Of the 10 prenatal RCTs, two reported conducting a subgroup analysis [25,52,77,78,94]. Subgroup analyses were conducted for sex [25,77,78], maternal education [94], maternal smoking during pregnancy [94], and stimulation in the home environment [52]. Subgroup analyses for sex showed poorer language scores, and greater risk of delayed language (score < 85) within girls only at 18 months of age [25], although no sex by treatment effects were detected in the assessments at 4 and 7 years of age [77,78]. Subgroup analyses with maternal education in the same trial revealed no effect of DHA supplements amongst women who had not completed tertiary education. However, lower language scores at 18 months of age were observed with DHA supplementation in women who had completed tertiary education [94]. Trial authors likewise explored whether there was a smoking (during pregnancy) by treatment effect, but found none [94]. In one trial, authors explored an interaction effect for quality of stimulation in the home environment during childhood [52]. For children in the DHA group, the home environment appeared to have less influence on developmental outcomes than for children in the control group [52].

#### 3.5.2. Assessments of Language Abilities after a DHA Intervention to Preterm Infants

The eight trials of preterm infant DHA supplementation showed no differences between treatment and control group language development in children between 9 months and 10 years of age, using 17 different assessments of language abilities [24,54,56,57,64,65,66,67,68,69,70,84,85].

(a) Four of the eight trials conducted in preterm infants included a clinician-administered global assessment with an overall language subscale [24,54,65,66,85], although none assessed global language. The Bayley-III was administered at 16–22 months of age in one trial [24], at 2–3 years in one trial [54], and at both 12 and 24 months of age in another trial [85], with no differences detected in either study. Two studies used the KPSDSI at 9 months and likewise detected no effect of the DHA intervention [65,66].
(b) Three of the eight RCTs in preterm infants included a parent-rated measure of child language [57,64,69]. Two RCTs included a general language measure completed by parents, the MCDI, administered at 9 months [64], 14 months [64], and at 26 months [69] of age. No evidence of a benefit of DHA intervention was detected in either trial, at any of the ages administered [64,69]. Parents completed a global measure with a communication subscale in one trial at 6 and again at 20 months of age, with no hint of an effect of DHA intervention at either age [57].
(c) Between the eight RCTs conducted in preterm infants, there were three that included a language-based cognitive assessment [67,70,84]. All trials administered the WASI, at 7 years [70], 8 years [84] and 10 years of age [67], with no effect of DHA supplementation on VIQ. One trial also conducted the NEPSY at the 10-year follow-up and likewise detected no benefit of the intervention [67].
(d) Two of the trials in preterm infants included an assessment of language-based academic abilities [67,70]. Both measured word reading and spelling in school-aged children and found no effect of the intervention [67,70]. Nor was there an effect on Pseudoword decoding [67].
(e) There were no assessments of language abilities not otherwise classified in the trials with preterm infant interventions.
(f) Of the eight preterm infant RCTs, 4 reported conducting a subgroup analysis [24,67,69,70,84] and two studies reported sensitivity analyses [64,67]. Subgroup and sensitivity analyses were conducted for sex [67,69,70,84], birthweight [24,69,70,84], and household income [24]. One of the earlier RCTs found a sex by treatment interaction where girls in the DHA group had better academic abilities, although there was no such effect on language-based cognitive abilities [67]. Performance of boys in this trial did not differ between groups [67]. In a larger trial including both breastfed and formula-fed preterm infants, subgroup analyses revealed no sex by treatment interaction on language development at 26 months [69], or 7 years [70] of age. Likewise, birthweight <1250 g or >1250 g did not appear to interact with language abilities in this same trial [69,70]. Another trial testing for interaction effects between sex and birthweight ≤1000 g found none [84]. A separate trial identified a negative effect of DHA supplementation on language at 16–22 months of age within infants with birthweight <1250 g [24]. This trial also explored household income and found no interaction effect with DHA supplementation on language outcomes [24]. Sensitivity analyses involving only the preterm infants who did not receive any breastmilk revealed benefits to language-based cognitive abilities in the intervention group at 10 years of age, but no effect on academic abilities [67]. Sensitivity analyses in another trial detected a benefit of the intervention to parent-rated vocabulary comprehension (although no effect on overall language scores, or language production) at 14 (but not 9) months when multiple births and non-English speaking families were excluded [64].

#### 3.5.3. Assessments of Language Abilities after Maternal Postnatal DHA Intervention

The three trials of postnatal maternal DHA supplementation primarily revealed null, but also some negative effects on seven comparisons of language development from 12 months to 5 years of age [32,53,62,63].

(a) One of the maternal postnatal trials conducted a clinician-assessment of language abilities [62], and one used a global development measure that included a language subscale [53]. No effect of the DHA intervention was detected with the CLAMS at 12 or 30 months of age [62]. Nor was an effect found on the Denver at 6–12 months, 12–18 months or at 18–24 months [53] of age.
(b) One trial in breastfeeding mothers administered the parent-rated MCDI at 12 and 24 months of age and found no group differences [32].
(c) One postnatal RCT in mothers administered a WPPSI-R at 5 years of age and detected no effect of DHA supplementation [63].
(d) No trials of DHA supplementation in breastfeeding mothers assessed language-based academic abilities.
(e) There were no assessments of language abilities not otherwise classified in the trials that supplemented breastfeeding mothers.
(f) Only one of the three maternal postnatal RCTs reported conducting a subgroup analysis for sex [32]. Authors found poorer language scores within boys in the DHA group, when compared with the control group, but no effect within females [32].

#### 3.5.4. Assessments of Language Abilities after Postnatal Infant DHA Intervention

The eight trials of infant DHA supplementation primarily revealed null effects on language development, as well as three instances of improvement and one instance of worse scores from 6 months to 10 years of age using 25 different assessments of language abilities [29,30,31,59,60,61,71,72,73,74,86,87,88,90].

(a) Of the eight trials conducted postnatally in infants, three included a clinician-administered assessment of global language abilities [29,31,73,89]. Null effects were reported for the CELF at 6 years [89] and the PPVT-R at 39 months [73] of age. However, in one trial with repeat language measures, DHA group children had a slightly worse PPVT-III score than control group children at 2 years, whilst there were no differences detected on the same assessment at 3.5 years [31] and at 5 years of age PPVT-4 scores were higher in the treatment group compared with the control group [29].

Five trials included overall language as a subscale of a global developmental test [29,30,74,86,87,90] and null effects were reported for the KPSDSI at 9 months [86], Bayley-III at 18 and 24 months [74,90], and Bayley-II at 18 months [87] of age, whilst another trial found that DHA group children had higher scores on the Bayley-II at 18 months of age [29,30].

(b) Of the eight RCTs in infants, only three included parent-rated measures of child language [29,71,72,74,89]. The MCDI was completed five times by parents at 9 months [72], 12 months [74], 14 months [71,72], and 18 months [29,74] of age with no hint of benefit of the DHA intervention at any age. The CCC was administered when children were 6 years of age [89] without any group differences. When the same children were 18 months of age, parents completed a language survey included in a behavior questionnaire [74] that likewise suggested no effect of DHA supplementation.
(c) From the 8 infant intervention trials there were seven IQ or cognitive tests [29,60,61,88]. The WASI was conducted at 9 years of age along with the NESPY in one trial where authors were testing for effect interactions with smoking [60]. There were 3 trials that administered the WPPSI test [29,61,88]. Two conducted a WPPSI-R at 4 years [88] and 6 years [61] of age, with no evidence of an effect of the intervention. However, in a third trial the WPPSI-III at 6 years of age detected higher scores in children who received the DHA intervention compared with children who received no DHA [29].
(d) One trial of infant DHA supplementation included an assessment of language-based academic abilities and school readiness at 2.5 years of age and found no differences between the groups in either the overall score, or subscales [31].
(e) Two infant intervention studies included an alternate/experimental assessment of language, and neither found a difference in randomization group performance on the task [73,89].
(f) Of the eight infant RCTs, two reported conducting a subgroup analysis [31,60], for sex [31], and for maternal smoking during pregnancy [60]. There was no evidence for a sex by treatment interaction for clinician-assessed language abilities at 2 years of age [31]. Subgroup analyses for prenatal smoking revealed a benefit of the DHA intervention to VIQ among children whose mothers smoked in pregnancy, but no effect among non-smokers [60].

## 4. Discussion

This is the first systematic review of the effect of DHA supplementation in early life on child language abilities. After conducting an exhaustive search strategy, we identified 29 eligible studies. There was a total of 84 language outcomes compared between a DHA and a control group, with 78 comparisons revealing no effect of a DHA intervention. We conclude that the existing evidence does not conclusively support or refute the hypothesis that DHA supplementation in the first 1000 days of life improves children’s language abilities. However, it is noteworthy that adequate DHA is likely to be important from conception through to 24 months of age, and that to date no trial has attempted to provide DHA across this entire period [2,7].

There was great heterogeneity in the timing and dose of DHA, although interventions were typically administered through capsules during pregnancy, lactation or infancy or infant formula for formula-fed infants. Language abilities were assessed with a wide variety of language measures at various ages, complicating comparability between studies and compounding the inappropriateness of combining results in a meta-analysis. Most assessments of language were in children up to 2 years of age (43 of 84 assessments), when language abilities are limited and still emerging. The majority of RCTs involved multiple longitudinal assessments of language abilities, with lack of group differences generally consistent at differing ages [25,26,27,28,29,30,31,32,56,57,58,62,63,64,66,67,68,69,70,71,72,73,74,75,76,77,78,83,84,87,88,89].

Of the 84 language assessments across 29 included RCTs, the majority reported no overall group differences [24,25,27,28,32,51,52,55,56,57,58,59,60,61,62,63,64,65,66,67,68,69,70,71,72,73,74,75,76,77,78,81,82,83,84,85,86,87,88,89,90]. Within trials where an effect of DHA supplementation was detected, effects were not identified consistently across all language outcomes [26,27,29,30,31,32,95].

It has previously been suggested that there may be participant characteristics that may modify the effect of a DHA intervention, and that RCTs should be encouraged to explore this possibility [94]. In particular, there is growing interest in the interaction effect of sex and early nutrition on outcomes [96]. However, there were only 9 of the 29 RCTs that explored the effect of DHA supplementation on language within a specific population subgroup. Evidence has pointed to the potential for inter-individual, biologically based, differences, such as child sex to moderate DHA synthesis [97,98,99,100,101]. There were six RCTs that reported subgroup analyses for sex [25,31,32,67,69,70,77,78,84], with no interaction detected in three trials [31,69,70,84], while two trials reported a difference within girls only [25,67] and one within boys only [32]. Exploration of birthweight by treatment interaction was considered in three trials of preterm infants, with no interaction effect of DHA reported in two [69,70,84], and a negative interaction effect in the other [24]. Trials that explored whether maternal education [94], maternal smoking during pregnancy [60,94], or home environment [24,52] had mixed findings. However, only three trials were likely powered to allow a meaningful subgroup analysis [25,51,52,68,70,77,78] and importantly, with all of these trials, the effects seen are generally in subgroup comparisons only, and could be Type I errors given that these are not the primary outcome and there are numerous comparisons. Further work is needed to determine whether there are any true characteristics that interact with DHA treatment, or whether there are subgroups of individuals more likely than others to respond positively to DHA supplementation.

DHA interventions are hypothesized to be beneficial for brain development, and at present there is no known mechanism for a negative effect of DHA. However, an optimal dose of DHA is yet to be identified, and it may be that exposure to excessive DHA is detrimental. In the formula trial testing three doses of DHA, the high-dose group performance was similar to or worse than the control group, while the low and mid-dose groups performed well [29,30,31]. In a trial of breastfeeding Danish women (who would naturally already be providing their infants with some DHA), increasing DHA intake led to some instances of worse language, mainly within boys, and infant DHA status at the end of the intervention period was negatively associated with vocabulary [32]. Of the studies that identified a possible negative effect of DHA on language, the doses were 800 mg/day or higher [25,32,77,78], and supplementation was in addition to DHA present in the diet of the participant. Additionally, two studies, one with subgroup analyses for maternal education and one exploring household income (both markers of socio-economic status likely positively associated with dietary DHA intake), indicate possible adverse effects of DHA supplementation in those who have completed higher levels of education or have higher household income [24,94]. Future research is needed to establish whether there is an upper limit for DHA intake, above which additional DHA is detrimental and whether DHA supplements should not be recommended for those already consuming sufficient dietary DHA.

Potential adverse effects of DHA supplementation are scarcely mentioned in other reviews and meta-analyses [19,20,21,22,23,40,41,42]. However, several intervention trials report negative effects of DHA, generally in subgroup analyses [24,25,70,78]. A prenatal trial found that parents of children in the DHA group perceived more behavioral problems and executive dysfunction at four and seven years of age than parents of children in the control group [25,77,78]. Similarly, in a RCT of DHA in neonates born <33 weeks’ gestation parents of females in the high-DHA group rated their children as having poorer behavioral functioning than parents of females in the standard-DHA group [70]. A RCT in toddlerhood for infants born preterm conducted subgroup analyses and found that among children from higher income households there was a possible negative effect of the DHA intervention on effortful control [24]. An infant study testing three formulas with varying doses of DHA to a control formula reported a consistent benefit at the two middle doses and a decline in performance on cognitive, language and executive functioning tasks in the group that received the highest dose of DHA, suggesting a dose-effect [29]. Importantly, with all of these trials, the effects seen are generally in secondary or exploratory outcomes in subgroup comparisons and could be Type I errors. There are currently no known mechanisms for DHA supplementation to have an adverse effect on brain development or functioning.

As a single nutrient intervention, DHA supplementation in nutritionally replete samples is likely to have a small-to-modest effect, requiring large samples in order to detect efficacy or adversity. This is particularly true if there are subpopulations that respond differently to increased DHA exposure. Furthermore, large samples increase the likelihood that characteristics conducive to optimal development, such as genetics and parental education, are balanced between the intervention and control groups, and hence will not confound group comparisons. Many of the trials included in this review had relatively small samples (*n* < 100 enrolled per group) [26,30,32,54,55,56,65,75,76,81,83,85,87,90] and were powered to detect relatively large differences rather than the modest effects that might be expected from a single nutrient intervention. Few trials attempted to account for possible confounders such as environmental stimulation [25,62,63,64,69,70,77,78], maternal intelligence quotient [63], paternal education [30,31,61,74] or maternal language [29], although all but one adjusted for maternal education [87,88]. Attrition was high (>20%) in many studies, and some attrition could be linked to post-randomization exclusions that could contribute to systematic loss to follow-up and attrition bias [93]. Given the already small and underpowered samples in many of the included trials, the chance of a Type I error may be increased.

Compounding the likelihood of a Type 1 error in the included studies is the fact that language was not a primary outcome of any trial, and all included trials compared multiple outcomes between the groups. A further important limitation is that few relevant DHA RCTs included a specific assessment of language abilities [24,26,27,28,29,32,64,69,71,72,73,75,76,82,83]. Several included trials only have a language score as part of an assessment of behavior [74,75], or general development [26,56,57,74], or from a cognitive assessment involving a measure of language-based cognitive abilities [52,55,58,65,66,67,70,75,76,84]. Several RCTs assessed language development using the MCDI [28,29,32,64,69,71,72,73,74,80], despite debate regarding its suitability for evaluating the effectiveness of interventions [102]. Two trials used the Bayley-I or II and attempted to calculate their own language score from the combined cognitive and language Mental Development Index [30,87]. Few trials provided scores for global language abilities with the majority using assessments that measured a specific domain, which for children under 24 months typically involves basic naming ability. Furthermore, there are numerous DHA trials that were not eligible for inclusion in this review as they had neither a language assessment nor assessment of language-based cognitive abilities, although they assessed an aspect of neurodevelopment such as cognition [51,103,104,105,106,107,108,109,110,111,112,113,114,115,116,117,118,119,120,121,122,123,124,125]. One trial was excluded as the form of supplementation was eggs (which contain DHA as well as other nutrients that contribute to neurodevelopment) and the control group received no intervention [126]. It is noteworthy that several ineligible RCTs used the Bayley-I or II (which combines cognition and language into a Mental Development Index) [29,51,57,59,62,64,65,66,68,71,86,87,103,111,113,118,119,120,121,127,128,129]. Hence, key aspects of language abilities may have been missed in many of RCTs assessing the effect of early DHA supplementation on child outcomes.

The effect of early DHA supplementation on language abilities as summarized here is comparable to previous reviews and meta-analyses of DHA interventions for child development outcomes [19,20,21,22,23,40,41,43]. Only one other review to date has synthesized DHA trials conducted during pregnancy, as well as for preterm infants, and postnatally [42]. Authors of this study reported child outcomes for cognition, motor and visual development across 38 trials, DHA intervention enhanced infant cognition (but not child IQ) and visual acuity [42]. This differs to the findings of previous high-quality reviews and meta-analyses, that considered only one period of supplementation (for example, during pregnancy only) [19,20,21,22,23,40]. Unlike our own review, the authors of this review did not report or detect any subgroup effects (for world region, race, maternal education, age at assessment, intervention duration or trial quality) [42]. However, they did not explore the subgroups considered in our current review, with the exception of maternal education. Importantly, the review also missed several trials and eligible follow-ups [24,60,70,77,78,82,83,85,90], particularly some of the key larger ones with null findings [24,60,70,77,78].

## 5. Conclusions

The present study provides the first overview of the totality of evidence around the effect of early DHA supplementation on the development of language. There were 84 assessments of language reported between 29 trials of DHA supplementation in the first 1000 days, with only four findings of a positive effect on a language outcome (three from the same RCT). Substantial variation between the included trials, such as the timing, duration, and dosage of the intervention, the sample (preterm or full-term, or both, and high or middle-to low-income countries) as well as range of language outcomes and assessments, and absence of consistent subgroup comparisons make it difficult to reach a definitive conclusion regarding the efficacy of early DHA interventions to improve language. Whilst the vast majority of trials consistently reported a null effect, two studies detected potentially adverse effects, suggesting that it may be worth considering the possibility of a detrimental effect of DHA exposure on language, at high doses and/or in specific subpopulations. This review supports the need to consider that blanket DHA supplementation strategies may not be appropriate. Further work is needed to identify whether there is an upper-limit for safe DHA exposure, and which (if any) population subgroups may benefit or be adversely effected by DHA supplementation.

## Figures and Tables

**Table 1:** Summary of the characteristics and results of randomized controlled trials included in the review.

Author and Reference	Setting Country; Recruitment	Participants Sample Size Special Characteristics	Intervention Duration, Form, Treatment and Control Intervention	Assessment Age, n, Outcome Measure	Result
Maternal prenatal interventions					
Dunstan 2008 [75], Meldrum 2015 [76]	Australia; antenatal clinic	*N* enrolled: 98 Trt: 52, ctrl: 46 all had allergic disease excluded: if normal diet included >2 fish meals/week	Duration: 20 w preg to birth Form: 4 capsules daily Trt: n-3 3300 mg/d, DHA 2200 mg/d Ctrl: olive oil	Age: 2.5 y, *n* = 72	
				PPVT	No diff
				GMDS	No diff
				CBCL	No diff
				Age: 12 y, *n* = 50	
				WISC-IV	No diff
				CCC-2	No diff
Karlson 2010 [55]	Sweden; antenatal clinic, local newspaper adverts	*N* enrolled: 145 Trt: 70, ctrl: 75 all had allergic disease Excluded: if taking n-3	Duration: 25 w preg to 3.5 mo Form: 9 capsules daily Trt: n-3 2700 mg/d, DHA 1100 mg/d Ctrl: soy oil	Age: 46 mo, *n* = 40	
				WPPSI-III	No diff
Makrides 2010 [25], Makrides 2014 [77], Gould 2017 [78]	Australia; antenatal clinic	*N* enrolled: 2399 Trt: 1197, ctrl: 1202 singletons Subset for neurodevelopmental follow-up *n* = 726 (preterm and randomly selected term)	Duration: 18–21 w preg to birth Form: 3x capsules daily Trt: 800 mg DHA/d Ctrl: vegetable oil	Age: 18 mo, *n* = 726	
				Bayley-III	No diff
				Age: 4 y, *n* = 646	
				CELF-P2	No diff
				DAS II	No diff
				Age: 7 y, *n* = 543	
				CELF-4	No diff
				WASI II	No diff
				WRAT-4	No diff
Ramakrishnan 2010 [51], Ramakrishnan 2016 [52]	Mexico; antenatal clinic	*N* enrolled: 1094 Trt: 547, ctrl: 547 Medium-low SES Excluded: if taking n-3	Duration: 18–22 w preg to birth Form: 2x capsules daily Trt: 400 mg/d DHA Ctrl: olive oil	Age: 5 y, *n* = 797	
				MSCA	No diff
	
Carlson 2013 [79], Colombo 2019 [80]	USA; antenatal clinics	*N* enrolled: 350 Trt: 178, ctrl: 172 Singleton, healthy, normal BMI	Duration: mean 14.5 w preg to birth Form: 3x daily capsules Trt: 600 mg/d DHA Ctrl: soy and corn oil	Age: 18 mo, *n* = 195	
				MCDI	No diff
				Age: 36 mo, *n* = 168	
				WPPSI-III	No diff
				Sentence repetition	No diff
				Age: 42 mo, *n* = 158	
				TOPEL	No diff
				Sentence repetition	No diff
				Age: 48 mo, *n* = 147	
				WPPSI-III	No diff
				Sentence repetition	No diff
				Age: 60 mo, *n* = 159	
				PPVT-3	No diff
				Age: 72 mo, *n* = 151	
				WPPSI-III	No diff
Mulder 2014 [27], Mulder 2018 [28]	Canada; NR	*N* enrolled: 270 Trt: 138, ctrl: 132 term-born singletons	Duration: 16 w preg to birth Form: daily capsules Trt: 400 mg/d DHA Ctrl: corn and soy oil	Age: 9 mo, *n* = 144	
				Recognition task	No diff
				Age 14 mo, *n* = 159	
				MCDI	No diff
				Age: 16 mo, *n* = 82	
				Word-Object pairing	No diff
				Age: 18 mo, *n* = 154	
				Bayley-III	No diff
				MCDI	No diff
				Age: 5–6 y, *n*= 97	
				PPVT	No diff
Keenan 2014 [81], Keenan 2016 [82]	USA; university medical centre	*N* enrolled: 64 Trt: 43, ctrl: 21 African- American women of low SES	Duration: 16–21 w preg to birth Form: 2x capsules daily Trt: 450 mg DHA + 90 mg EPA/day Ctrl: corn and soybean oil	Age: 3 mo, *n* = 49	
				Bayley-III	No diff
Miller 2016 [83]	USA; antenatal clinic	*N* enrolled: 115 Trt: 60, ctrl: 55 singletons	Duration: 24–28 w preg to 3 mo Form: capsules Trt: 300 mg DHA + 67 mg EPA/d Ctrl: sunflower oil	Age: 4 mo, *n* = 91	
				Bayley-III	No diff
				Age: 12 mo, *n* = 83	
				Bayley-III	No diff
Ostradrahimi 2017 [26]	Iran; healthcare centres	*N* enrolled: 150 Trt: 75, ctrl: 75	Duration: 20 w preg to 30 days Form: capsules Trt: 120 mg DHA + 180 mg EPA/day Ctrl: liquid paraffin	Age: 4 mo, *n* = 148	Trt
				ASQ	improved
				Age: 6 mo, *n* = 146	
				ASQ	No diff
Brei 2017 [58]	Germany; NR	*N* enrolled: 208 Trt: 104, ctrl: 104	Duration: 15 w preg to 4 mo Form: capsules (+ dietary counselling to ↓ AA intake) Trt: 1020 mg DHA + 180 mg EPA/day Ctrl: general dietary information	Age: 4 y, *n* = 119	
				CDI	No diff
				Age: 5 y, *n* = 130	
				CDI	No diff
Interventions for preterm infants (born <37 weeks’ gestation)					
O’Connor 2001 [64]	USA, UK, South America; neonatal intensive care units	*N* enrolled: 470 Trt1: 140, Trt2: 143 ctrl: 144 birthweight 750–1800 g singletons and multiples <33 w FF or BF	Duration: <72 h of enteral feeding to 12 mo Form: formula (infants also receiving breastmilk) Trt1: DHA + AA from fish/fungal oil Trt2: DHA + AA from egg Ctrl: no DHA	Age: 9 mo	
				MCDI	No diff
				Age: 14 mo	
				MCDI	No diff
Fewtrell 2002 [65]	UK; neonatal units	*N* enrolled: 195 Trt: 95, ctrl: 100 <37 w, birthweight <1750 g, FF	Duration: <10 days until discharge Form: Formula Trt: LCPUFA formula Ctrl: standard formula	Age: 9 mo *n* = 158	
				KPSDSI	No diff
Fewtrell 2004 [66], Isaacs 2011 [67]	UK; neonatal units	*N* enrolled: 238 Trt: 122, ctrl: 116 <35 w, birthweight ≤2000 g FF	Duration: before discharge to 9 mo CA Form: formula Trt: LCPUFA formula Ctrl: standard formula	Age: 9 mo CA *n* = 117	
				KPSDSI	No diff
				Age: 10 y *n* = 107	
				WASI	No diff
				NEPSY	No diff
				WIAT-II	No diff
Henriksen 2008 [56], Westerberg 2011 [57], Almaas 2015 [84]	Norway; neonatal units	*N* enrolled: 141 Trt: 68, ctrl: 73 birthweight < 1500 g BF only	Duration: from enteral feeds to discharge from hospital or infant finished the 100 mL bottle of oil Form: oil added to breast milkTrt: 32 mg DHA + 31 mg AA/100 mL breastmilk Ctrl: soy oil	Age: 6 mo CA *n* = 105	
				ASQ	No diff
	
				Age: 20 mo CA *n* = 92	
				ASQ	No diff
				Age: 8 y *n* = 98	
				WASI	No diff
Makrides 2009 [68], Smithers 2010 [69], Collins 2015 [70]	Australia; neonatal units	*N* enrolled: 657 Trt: 322, ctrl: 335 singletons and multiples <33 wsmall subset for follow-up at 26 mo FF and BF	Duration: <5 d of starting full enteral feeds to term equivalent Form: Preterm infant formula, 6x capsules daily to breastfeeding mothers Trt: Formula 1% DHA Ctrl: Formula 0.35% DHA	Age: 26 mo CA *n* = 128	
				MCDI	No diff
				Age: 7 y CA *n* = 604	
				WASI	No diff
				WRAT-4	No diff
Keim 2018 [24]	USA; neonatal intensive care units	*N* enrolled: 377 Trt: 189, ctrl: 188 singletons and multiples <35 w, no longer FF or BF	Duration: 10–16 mo CA for 6 mo Form: dissoluble powder Trt: 200 mg DHA + 200 mg AA/d Ctrl: 400 mg corn oil/d	Age: 16–22 mo *n* = 377	
				Bayley-III	No diff
Andrew 2018 [85]	UK; neonatal units	*N* enrolled: 59 Trt: 29, ctrl: 30 Singletons <31 w/with risk of neurodevelopmental impairment (such as brain injury)	Duration: from full milk feeds for 2 y Form: sachet to mix with milk or food Trt: DHA 1% fatty acids Ctrl: no DHA	Age: 12 mo, *n* = 45	
				Bayley-III	No diff
				Age: 24 mo, *n* = 43	
				Bayley-III	No diff
Hewawasam 2020 [54]	Australia; neonatal units	*N* enrolled: 192 Trt: 96, ctrl: 96 <29 w with no major congenital or chromosomal abnormality	Duration: <3 d of starting full enteral feeds to term equivalent Form: enteral emulsion Trt: 60 mg/kg/day DHA Ctrl: no DHA	Age: 2–3 y, *n* = 77	
				Bayley-III	No diff
	
Postnatal interventions for breastfeeding mothers					
Lauritzen 2005 [32]	Denmark; antenatal general practitioner visit (via Danish National Birth Cohort)	*N* enrolled: 175 Trt: 62, ctrl: 60, Ref: 53 Healthy term infants BF habitual fish intake below Danish median	Duration: <7 days for 4 mo Form: muesli bars, cookies and capsules Trt: 4.6 g fish oil, 1.5 g LCPUFA Ctrl: no DHA Ref: high habitual fish intake	Age: 12 mo, *n* = 131	
				MCDI	Trt worse
				Age 24 mo, *n* = 111	
				MCDI	No diff
Jensen 2005 [62], Jensen 2010 [63]	USA; advertising in newspaper, physicians’ offices, childbirth classes	*N* enrolled: 230 Trt: 115, ctrl: 115 BF term infants singletons and twins	Duration: < 5 days for 4 mo Form: capsules Trt: 200 mg DHA Ctrl: vegetable oil	Age:12 mo, *n* = 162	
				CLAMS	No diff
				Age:30 mo, *n* = 160	
				CLAMS	No diff
				Age:5 y, *n* = 119	
				WPPSI-R	No diff
Argaw 2018 [53]	Ethiopia; NR	*N* enrolled: 360 Trt1: 90, Trt2: 89, Trt3: 90, ctrl: 91 BF healthy singletons	Duration: 6–12 mo for 12 mo Form: Mother-capsules, Child-complimentary food supplements Trt1: Mother-215 mg DHA + 285 mg EPA, Child-169 mg DHA + 331 mg EPA Trt2: Mother-215 mg DHA + 285 mg EPA Trt3: Child-169 mg DHA + 331 mg EPActrl: Mother-corn oil, Child-corn + soy oil	Age: Baseline, 6–12 mo, *n* = NR	
				Denver	No diff
				Age: after 6 mo, 12–18 mo, *n* = 326	No diff
				Denver	
				Age: after 12 mo, 18–24 mo, *n* = 313	
				Denver	No diff
Postnatal interventions for infants					
Scott 1998 [71], Auestad 2001 [72], Auestad 2003 [73]	USA; children’s hospital	*N* enrolled: 404 Trt1: 82, Trt2: 80, ctrl: 77, BF: 165 healthy term bornFF	Duration: <7 days to 12 mo Form: formula Trt1: 0.13% DHA-egg Trt2: 0.13% DHA-fish/fungal Ctrl: no LCPUFA BF: Trt1 & Trt2 formula if stopped BF	Age 9 mo, *n* = 163	
				MCDI	No diff/no overall score
				Age: 14 mo, *n* = 173	
				MCDI	No diff/no overall score
				Age 39 mo, *n* = 157	
				PPVT-R	No diff
				MLU	No diff
Lucas 1999 [86]	UK; hospital	*N* enrolled: 447 Trt: 155, ctrl: 154, BF: 138 term born FF	Duration: <7 days to 6 mo Form: formula Trt: 0.32%DHA Ctrl: no DHA	Age: 9 mo *n* = 241 + BF = NR	
				KPSDSI	No diff
Willatts 2013 [61]	UK, Belgium, Italy; antenatal clinic	*N* enrolled: 376 Trt: 126, ctrl: 111, BF: 139 FF	Duration: <7 days to 4 mo Form: formula Trt: 0.30% DHA Ctrl: no DHA	Age:6 y, *n* = 235	
				WPPSI-R	No diff
Birch 2000 [87], Birch 2007 [88]	USA; hospitals	*N* enrolled: 119 Trt1: 26, Trt2: 27, ctrl: 26, BF: 40 healthy term-born FF	Duration: <5 days to 17 w Form: formula Trt1: 0.35% DHA Trt2: 0.36% DHAC trl: no DHA BF: no formula	Age: 18 mo, *n* = 76	
				Bayley-II	No diff
				Age: 4 y, *n* = 84	
				WPPSI-R	No diff
Bouwstra 2005 [59], de Jong 2012 [60]	Netherlands; antenatal clinics	*N* enrolled:474 Trt: 145, ctrl: 169, BF: 160 healthy term-born FF	Duration: 2 mo to 6 mo Form: formula Trt: 0.3% DHA + 0.45%AA Ctrl: no DHA BF: Trt formula if stopped BF	Age: 9 y, *n* = 341	
				WASI	NR
				NEPSY	No diff
Drover 2011 [30], Drover 2012 [31], Colombo 2013 [29]	USA; hospitals	*N* enrolled: 159 Trt1: 38, Trt2: 39, Trt3: 40, ctrl: 42 healthy full-term singletons low SES FF	Duration: 1–9 days to 12 mo Form: formula Trt1: 0.32% DHA Trt2: 0.64% DHA Trt3: 0.96% DHA Ctrl: no DHA	Age: 18 mo, *n* = 92	No diff
				MCDI	
				Bayley-II	Trt improved
				Age:2 y, *n* = 99	
				PPVT-III	Trt worse
				Age 2.5 y, *n* = 93	
				BBCS-R	No diff
				Age:3.5 y, *n* = 88	
				PPVT-III	No diff
				Age:5 y, *n* = 81	
				PPVT-4	Trt improved
				Age: 6 y, *n* = 81	
				WPPSI-III	Trt improv ed
Meldrum 2012 [74], Meldrum 2020 [89]	Australia; antenatal clinic	*N* enrolled: 420 Trt: 218, ctrl: 202 All mothers had allergic disease FF and BF excluded preterm	Duration: birth to 6 mo Form: oil capsules Trt: 250–280 mg/d DHA Ctrl: olive oil	Age: 12 mo, *n* = 128	
				MCDI	No diff
				Age:18 mo, *n* = 287	
				Bayley-III	No diff
				MCDI (*n* = 269)	No diff
				CBCL (*n* = 185)	No diff
				Age: 6 y, *n* = 304	
				CELF-4	No diff
				Renfrew Bus Story	No diff
				CCC-2	No diff
Devlin 2017 [90]	Canada; community advertising, immunization clinics	*N* enrolled: 133 Trt: 68, ctrl: 65 Term, singleton, normal birthweight, BF < twice/d, English primary language	Duration: 12–14 mo ± 7 d to 24 mo Form: sprinkles x2/d Trt: 200 mg/d DHA Ctrl: corn oil	Age: 24 mo, *n* = 110	
				Bayley-III	No diff

AA = Arachidonic Acid; ASQ = Ages and Stages Questionnaire (subscale-communication); Bayley = Bayley Scales of Infant Development (edition III has a standardized global Language Scale score, editions I and II combine cognitive and language into one scale although some authors attempt to calculate a language score); BBCS = Bracken Basic Concept Scale (receptive language and acquisition of basic concepts); BF = breastfed; CA = corrected age (corrected for prematurity); BMI=body mass index; CBCL = Child Behavior Checklist (subscale-Language Development Survey, parent-rated number of words in infant vocabulary); CCC = Children’s Communication Checklist; CDI = Child Development Inventory (subscales-Expressive Language, Language Comprehension, Letters); CELF = Clinical Evaluation of Language Fundamentals (P = Preschool edition); CLAMS = Clinical Linguistic and Auditory Milestone Scale; Ctrl = control group; d = day(s); DAS = Differential Ability Scales (subscale-Verbal Scale Score); Denver = Denver Developmental Screening Test (subscale-language); DHA = Docosahexaenoic acid; EPA = Eicosapentaenoic acid; FF = formula fed; GMDS = Griffiths Mental Development Scale (subscale-Speech and Hearing subscale); KPSDSI = Knobloch, Passamanick and Sherrard’s Developmental Screening Inventory (Subscale-Language); LCPUFA = Long-chain polyunsaturated fatty acid; MCDI = MacArthur-Bates Communicative Development Inventories; MLU = Mean length of utterances during free-play with parent; mo = month(s); MSCA = McCarthy Scales of Children’s Abilities (subscale-Verbal); NEPSY = Developmental NEuroPSYchological Assessment (subscale-language domain); No diff = No difference; NR = Not reported; n-3 = Omega-3 long chain polyunsaturated fatty acid; preg = pregnancy; PPVT = Peabody Picture Vocabulary Test; R = revised; Ref=reference group; SES= socioeconomic status; TOPEL = Test of Preschool Early Literacy (vocabulary, phonological awareness, print knowledge); Trt = treatment group; UK = United Kingdom; USA = United States of America; WASI = Wechsler Abbreviated Scale of Intelligence (subscale-VIQ); WIAT = Wechsler Individual Achievement Test (subscale-Word Reading, Spelling, Pseudoword decoding); WISC = Wechsler Intelligence Scale for Children (subscale-VIQ); w = week(s); WPPSI = Wechsler Preschool and Primary Scale of Intelligence (subscales-VIQ); WRAT = Wide Range Achievement Test (subtests-Word Reading, Spelling); y= year(s); ↓= decreased.
